# Dopamine Depletion Affects Vocal Acoustics and Disrupts Sensorimotor Adaptation in Songbirds

**DOI:** 10.1523/ENEURO.0190-19.2019

**Published:** 2019-06-11

**Authors:** Varun Saravanan, Lukas A. Hoffmann, Amanda L. Jacob, Gordon J. Berman, Samuel J. Sober

**Affiliations:** 1 Neuroscience Graduate Program, Graduate Division of Biological and Biomedical Sciences, Laney Graduate School, Emory University, Atlanta, GA 30322; 2 Department of Biology, Emory University, Atlanta, GA 30322; 3 Department of Physics, Emory University, Atlanta, GA 30322

**Keywords:** basal ganglia, Bengalese finch, dopamine, sensorimotor adaptation, songbird, vocal learning

## Abstract

Dopamine is hypothesized to convey error information in reinforcement learning tasks with explicit appetitive or aversive cues. However, during motor skill learning feedback signals arise from an animal’s evaluation of sensory feedback resulting from its own behavior, rather than any external reward or punishment. It has previously been shown that intact dopaminergic signaling from the ventral tegmental area/substantia nigra pars compacta (VTA/SNc) complex is necessary for vocal learning when songbirds modify their vocalizations to avoid hearing distorted auditory feedback (playbacks of white noise). However, it remains unclear whether dopaminergic signaling underlies vocal learning in response to more naturalistic errors (pitch-shifted feedback delivered via headphones). We used male Bengalese finches (*Lonchura striata* var. *domestica*) to test the hypothesis that the necessity of dopamine signaling is shared between the two types of learning. We combined 6-hydroxydopamine (6-OHDA) lesions of dopaminergic terminals within Area X, a basal ganglia nucleus critical for song learning, with a headphones learning paradigm that shifted the pitch of auditory feedback and compared their learning to that of unlesioned controls. We found that 6-OHDA lesions affected song behavior in two ways. First, over a period of days lesioned birds systematically lowered their pitch regardless of the presence or absence of auditory errors. Second, 6-OHDA lesioned birds also displayed severe deficits in sensorimotor learning in response to pitch-shifted feedback. Our results suggest roles for dopamine in both motor production and auditory error processing, and a shared mechanism underlying vocal learning in response to both distorted and pitch-shifted auditory feedback.

## Significance Statement

Dopamine has been hypothesized to convey a reward prediction error signal in learning tasks involving external reinforcement. However, the role dopamine plays in tasks involving self-guided error correction in the absence of external reinforcement is much less clear. To address this question, we studied the role of dopamine in sensorimotor adaptation using male Bengalese finches, which spontaneously produce a complex motor behavior (song) and are capable of modulating their behavioral output in response to induced auditory errors. Our results reveal that in addition to conveying errors in motor performance, dopamine may also have a role in modulating effort and in choosing a corrective response to the auditory error.

## Introduction

Complex organisms perform sensorimotor learning to modulate behavior in response to sensory feedback. This process uses feedback from past performances arising from either explicit reward/punishment cues (e.g., food reward, electric shocks) or from self-evaluation of the performance (e.g., hearing one’s own voice during speech or song). While prior work has taken a number of approaches to taxonomizing different forms sensorimotor learning, including distinguishing model-based and model-free learning (Wolpert et al., 1995; Mohan et al., 2011; Haith and Krakauer, 2013) and habitual versus goal-directed behavior (Balleine and O'doherty, 2010; Redgrave et al., 2010), here we focus on an orthogonal distinction into two broad components: error-based learning that relies on self-evaluation and reinforcement learning that relies on cues from the environment (Wolpert et al., 2011). Classic studies have linked dopamine to reinforcement learning as a reward prediction error signal that conveys information about explicit rewards and punishments (Schultz et al., 1997; Glimcher, 2011). However, the question of whether dopamine is also involved in error-based learning in the absence of external rewarding or aversive cues has been harder to address. Some studies have reported deficits in error-based learning in patients with Parkinson’s disease (Paquet et al., 2008; Mollaei et al., 2013), but since Parkinson’s disease is associated with cognitive and executive deficits in addition to larger motor deficits (Lees and Smith, 1983; Cooper et al., 1991; Dubois and Pillon, 1996; Jankovic, 2008), the specific role of dopamine has been difficult to isolate.

**Figure 1.**
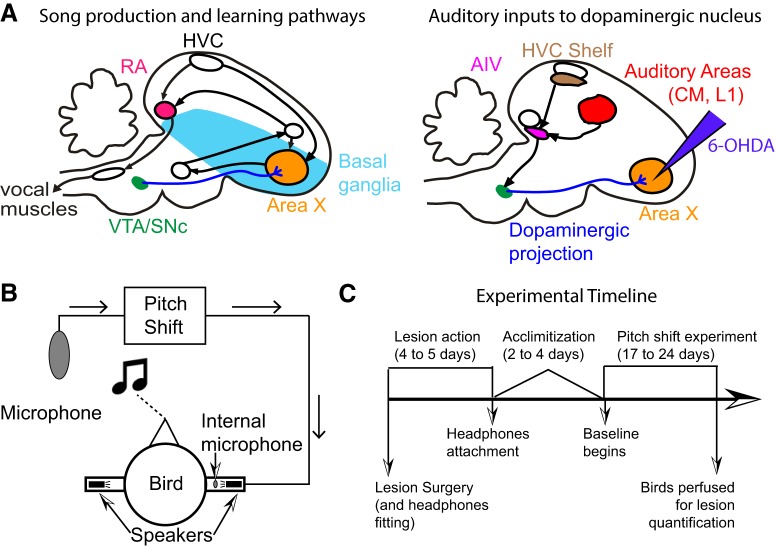
Songbird neuroanatomy and experimental design. ***A***, A theory for the role of dopamine in sensorimotor learning in songbirds. The left panel shows the brain nuclei in the songbird primarily involved in song production and learning. Area X, a songbird basal ganglia nucleus critical for song learning, receives dense dopaminergic projections from the VTA/SNc complex. The right panel shows the nuclei involved in auditory processing in the songbird. There are other inputs (data not shown) to the VTA/SNc complex from auditory areas and the ventral basal ganglia (vBG). One of the known pathways for auditory information to influence song learning is through the dopaminergic projections to Area X. We target these projections when we perform 6-OHDA lesions into Area X as depicted. ***B***, A schematic for how the custom-built headphones introduce a pitch shifted auditory error to the birds. Briefly, a cage microphone records all sounds made within the cage and sends it through a pitch shifting program which is subsequently played back to the bird through miniature speakers attached to the headphones. The headphones also have an internal microphone to record output from the headphones speakers and to calibrate sound intensity. ***C***, A detailed timeline for each of our experiments (see Materials and Methods).

Songbirds have emerged as an effective model system in which to study the role of dopamine in sensorimotor learning. Songbirds spontaneously produce songs hundreds of times per day. Like human speech, song is learned during development (Wilbrecht and Nottebohm, 2003; Lipkind et al., 2013) and actively maintained by auditory feedback through adulthood (Sakata and Brainard, 2006, 2008; Sober and Brainard, 2009; Kuebrich and Sober, 2015). Additionally, songbirds have a well-defined neural circuitry dedicated to song production and song learning (Sohrabji et al., 1990; Scharff and Nottebohm, 1991; Brainard and Doupe, 2000). Dopaminergic neurons from the ventral tegmental area/substantia nigra pars compacta (VTA/SNc) complex innervate Area X, a basal ganglia nucleus essential for song learning, and have been hypothesized as a way for auditory error information to enter the song system (Bottjer, 1993; Soha et al., 1996; Mandelblat-Cerf et al., 2014; Peh et al., 2015; Fig. 1). Researchers examining vocal control employ two primary methods to induce song learning in adult songbirds: through distorted auditory feedback (Tumer and Brainard, 2007) and through pitch shifts played through custom-made headphones (Sober and Brainard, 2009). It remains unclear to what extent the two paradigms share underlying neural mechanisms. Dopamine has been shown to be involved in changing the pitch of the song in response to distorted auditory feedback. Specifically, birds display deficits in learning to avoid distorted feedback under dopamine depleted conditions (Hoffmann et al., 2016; Hisey et al., 2018), neural recordings of dopaminergic neurons revealed prediction error type responses when birds were required to avoid such auditory distortions while singing (Gadagkar et al., 2016), and pitch-contingent optical stimulation of dopaminergic terminals in Area X evoked changes in the pitch of the birds’ song (Hisey et al., 2018; Xiao et al., 2018). Here, we tested the hypothesis that there are common neural mechanisms underlying both learning paradigms by studying the role of dopamine in birds when they respond to a pitch shifted version of their own auditory feedback (Sober and Brainard, 2009).

We tested the role of dopamine in error-based learning by selectively lesioning dopaminergic terminals in Area X using 6-hydroxydopamine (6-OHDA). Since the cell bodies of dopaminergic neurons in VTA/SNc that innervate Area X are intermingled with those projecting to the rest of the songbird basal ganglia (Person et al., 2008), we injected 6-OHDA directly into Area X to avoid introducing general motor or song production deficits. We fitted the birds with custom-built headphones through which we introduced a shift in pitch (either upwards or downwards) of the bird’s auditory feedback (Sober and Brainard, 2009; Hoffmann et al., 2012) to measure how birds changed their pitch over time in response to this induced sensory error and how self-guided error correction was affected by dopamine manipulations.

## Materials and Methods

All animals used for this study were adult (range of ages: 105–217 days post hatch; median age: 141 days post hatch) male Bengalese finches (*Lonchura striata* var. *domestica*). Throughout the study, the animals were housed in isolated sound attenuating chambers (referred to as sound boxes) on a 14/10 h light/dark cycle. All singing analyzed for this paper was undirected song, i.e., songs sung in the absence of a female. All procedures were approved by Emory University’s Institutional Animal Care and Use Committee.

### Experimental design

Songbirds display significant bird-by-bird variability in amount of learning displayed, and so most experimental designs include a within-bird control to measure the amount of learning within a bird before and after a manipulation of interest (Hoffmann et al., 2016; Hisey et al., 2018). However, in the case of headphones as we use here (described in Headphones Attachment and Assembly below), the only way to secure the headphones to the birds for the duration of the experiment is to cement them to the skull. Although this method ensures that the headphones fit comfortably around the ear canals and remain in place for the duration of the experiment, cementing the headphones to the skull prevents access to the brain, thereby preventing us from examining learning in the same animals before and after lesion. As a result, we designed a group comparison study to test the role of dopamine in sensorimotor adaptation. We performed pitch shift experiments on six unlesioned birds (three each for upward shifts and downward shifts) and eight lesioned birds (four for upward pitch shift and four for downward pitch shift). As detailed below, virtual auditory feedback through the headphones was delivered almost in real time and was meant to replace the natural auditory feedback that birds would otherwise receive. All pitch shifts were one semitone in magnitude (equally split between +1 and –1 semitone shifts). Each experiment consisted of 3 d of baseline (unshifted auditory feedback through headphones) followed by 14 d of pitch shifted auditory feedback. At the end of the shift period, we turned off the shift in pitch (i.e., set the pitch shift to zero semitones as in the baseline epoch) and recorded the birds’ activity for 6–7 d. During this period, unlesioned birds typically reverse the effects of the pitch shift (Sober and Brainard, 2009). We refer to this period as “washout.” Washout data were collected for all six unlesioned birds. Due to technical difficulties associated with keeping the headphones attached for extended periods of time, washout data were collected for only four out of the eight lesioned birds (two for upward pitch shifts and two for downward). In addition, we performed control experiments with two unlesioned birds fitted with headphones and no pitch shift and eight lesioned birds without any pitch shifts (five with headphones and zero pitch shift throughout; three with no headphones). To minimize the number of animals we used, our unlesioned bird group consisted of data reanalyzed from Sober and Brainard, 2009. All data that have not been labeled explicitly as “data reanalyzed from a previous study” are new data collected for the purpose of this study. Furthermore, since we showed previously (Hoffmann et al., 2016) that animals injected with saline instead of 6-OHDA were statistically indistinguishable from unlesioned birds, we did not include a saline-injected control group in this study. Note that of the eight birds whose data were reanalyzed from Sober and Brainard (2009), the raw data for two animals, the unlesioned birds with no headphones shift, were unavailable. However, we were able to extract the daily mean pitch values from each animal’s data from an eps version of the original figure summarizing the data. The resulting figure that shows the mean change in pitch and error bars for the group was produced from the two data points for each day.

For our lesioned group, we reduced the dopaminergic innervation of Area X (Fig. 1), a song specific nucleus of the basal ganglia, using 6-OHDA microinjections as described in detail previously (Hoffmann et al., 2016). Following 6-OHDA surgery, the birds were allowed to recover in their sound boxes for 4–5 d. This coincides with the period over which 6-OHDA is known to cause degeneration of striatal innervation (Jeon et al., 1995). Subsequently, the headphones (Hoffmann et al., 2012) were fitted to the birds and set to initially provide unshifted auditory feedback (zero pitch shift). Following headphones attachment, the birds typically did not sing for 2–4 d (for a timeline schematic, see Fig.1*C*). Once they started singing again (defined as at least 30 song bouts produced over the entire day), we began recording a 3-d baseline period. Following the 3 d of baseline, the birds were recorded for 14 d during a period of shift. As described previously (Sober and Brainard, 2009; Kelly and Sober, 2014), the pitch shift was a one semitone shift (either upwards or downwards) played back to the bird through the headphones. The auditory feedback through the headphones was almost real time (delay of ∼10 ms) and was intended to replace the bird’s natural auditory feedback. In order to do so, the volume is set to be at least 2 log units greater in sound intensity than the bird’s own feedback. For the birds that had no pitch shift through the headphones, they continued with zero shift as they were in baseline for the equivalent 14 d. Following this 14-d period, we recorded the birds’ activity for 6–7 d of washout. Owing to the difficulties of keeping the headphones attached and functional for long periods of time, we were not able to collect washout data for every animal. Analysis of washout was therefore necessarily limited to birds that did have data collected for the washout period.

Note that one of our 6-OHDA lesioned birds in the –1 semitone shift group was subjected to an extended baseline period of 6 d rather than the 3-d period used for all other animals. Excluding data from this bird did not change any of our results significantly. Therefore, all results reported include this bird, treating the last 3 d of baseline equivalent to days 1 through 3 of baseline for every other bird.

Birds with lesions that were not fitted with headphones were returned to their sound boxes postsurgery and were recorded for the duration of the experiment. In this case, since they did not have a break in singing due to placement of fully assembled headphones, the baseline was defined as days 6 through 8 after lesion and the “shift” period was defined as day 9 through 22 after lesion to keep the timelines comparable between groups.

### 6-OHDA lesions

We performed the lesions using stereotactic surgeries as described in detail previously (Hoffmann et al., 2016). Briefly, birds were anesthetized using ketamine and midazolam and positioned at a beak angle of 20° below horizontal. Isoflurane was used to sustain anesthesia following the first hour of surgery. All stereotactic coordinates were relative to the landmark Y_0_, the posterior border to the divergence of the central sinus in songbirds. Small craniotomies were performed above the coordinates AP 4.75–6.4 mm; ML 0.75–2.3 mm on both sides. 6-OHDA (Tocris; conjugated with HBr) was injected bilaterally in a 4 × 3 mm grid at AP coordinates 5.1, 5.5, 5.9, and 6.3 mm and ML coordinates 0.9, 1.55, and 2.2 mm with a DV coordinate between 3.08 and 3.18 mm from the surface of the brain. For each injection, the glass pipette was lowered into the brain slowly allowing for time for rebounding of tissue, and following the injection, the pipette was left in place for at least 30 s before withdrawal at a similarly slow pace. Additionally, we initially performed one final injection at AP 4.8 mm, ML 0.8 mm, and DV 2.6 mm from the surface of the brain targeting the tail portion of Area X but dropped this injection in later birds as the targeting was not reliable and the injection required a larger craniotomy to perform. 13.8 nl of 6-OHDA was injected in the slow setting (23 nl/s) at each injection site using a Drummond Scientific Nanoject II auto-nanoliter injector.

### Headphones attachment and assembly

The methodology is described in detail in Hoffmann et al. (2012). Briefly, each set of headphones was custom-fit to an individual bird under anesthesia. If attached on a bird that also had a 6-OHDA lesion, both lesion and headphones fit adjustment were performed back-to-back in the same surgery. Once the headphones had been successfully fitted for the bird, the electronics (a speaker on each side and a miniature microphone on one side to record headphones output and calibrate volume) were assembled offline. The fully assembled headphones were then refitted to the bird 4–5 d after surgery. We used a flexible tether with a commutator to power the headphones and read the electronic signals.

### Histology

Following the end of the experiment, headphones were removed and the birds were deeply anesthetized with ketamine and midazolam before performing perfusions using 10% formalin. The brains were postfixed overnight in formalin and then cryoprotected in 30% sucrose for 1–4 d before slicing into 40 µm sections on a freezing sliding microtome. Alternating sections were either immunoreacted with tyrosine hydroxylase (TH) antibody and visualized with diaminobenzidine (TH-DAB) or Nissl-stained. TH-DAB was used to quantify the extent of lesions in the 6-OHDA birds, while Nissl was used to verify that there had been no necrosis and to assist in identifying boundaries of Area X in adjacent TH-DAB sections. For the TH-DAB reaction, all incubations were conducted on a shaker at room temperature and all chemicals were dissolved in 0.1 M phosphate buffer (PB) unless otherwise noted. Fixed sections were treated sequentially with 0.3% hydrogen peroxide to suppress endogenous peroxidases and 1% sodium borohydride to reduce exposed aldehydes and improve background staining before incubating overnight in a TH antibody solution (Millipore catalog #MAB318, RRID:AB_2201528, 1:4000; 0.3% Triton X-100; and 5% normal horse serum). Tissue was then incubated in biotinylated anti-mouse secondary antibody (Vector Laboratories catalog #BA-2000, RRID:AB_2313581, 1:200 and 0.3% Triton X-100) followed by avidin-biotin-complex (ABC) solution (Vector Laboratories catalog #PK-4000, RRID:AB_2336818). Tissue was exposed to DAB solution (Amresco E733; 5 mg DAB per tablet; two tablets in 20 ml of purified water) for ∼5 min. Sections were mounted, air-dried, delipidized with ethanol and citrisolv, and coverslipped with Permount (Fisher Scientific, SP15-500). For the Nissl-stained sections, Nissl stain was applied on mounted, air-dried tissue, which was delipidized with ethanol and citrisolv, and coverslipped with Permount. Stained sections were imaged using a slide scanner (Meyer Instruments PathScan Enabler IV; 24-bit color, 7200 dpi, “sharpen more” filter, brightness, and contrast level 50) and the resulting images were analyzed using ImageJ (RRID:SCR_003070).

### Image and lesion analysis

TH-DAB-stained sections were used for lesion quantification by analysis through a custom written macro in ImageJ. The analysis was based on a metric of optical density (OD) described in detail in Hoffmann et al. (2016). Briefly, the macro allowed us to demarcate the boundary of Area X in every section that it is present. We also used a circle of diameter 0.5 mm to mark a section of representative striatum outside of Area X in the same section. We then defined the OD ratio as the ratio between the OD of Area X in the section to that of striatum in the section as follows:

$$ODratio=\frac{OD_{AreaX}}{OD_{striatum}}$$

One of the established ways of identifying Area X in songbirds has been that Area X is darker than the surrounding striatum when stained with TH-DAB (Bottjer, 1993; Soha et al., 1996; Hoffmann et al., 2016). Due to this property, we used the cumulative distribution of the OD ratio in saline-injected birds to define our threshold for lesions. Any section in our group of 6-OHDA lesioned birds with an OD ratio less than the 5th percentile of the saline-injected birds sections counted toward the overall proportion of lesioned sections. Additionally, we used a two-sample Kolmogorov–Smirnov test to test whether the lesioned and saline populations were indeed drawn from separate distributions. We also used the threshold procedure described above to quantify lesion extent for individual animals. We then asked whether lesion extent was significantly correlated with vocal behavior metrics such as baseline variance, change in variance from baseline to end of shift and change in pitch at the end of shift. Note however that while this metric is robust at the population level, it is less so for individual birds.

### Pitch quantification

All our analysis was performed using an extracted value of pitch for every instance in which a bird sings a particular syllable. Briefly, birds have multiple syllables within their song and they typically repeat their song hundreds of times per day during the course of the experiment. We call each time they sing a particular syllable an iteration of that syllable. We restricted our analysis to roughly 30 song files per day between 10 A.M. to 12 P.M. and have shown earlier that the choice of time window does not qualitatively affect our results (Sober and Brainard, 2009; Hoffmann and Sober, 2014; Kelly and Sober, 2014). To quantify pitch, for each syllable we specify a time during the syllable (relative to syllable onset) during which the syllable is relatively flat and clear in the frequency versus time space and can be reliably quantified across iterations across days. The pitch we extract represents a weighted average of the frequencies with the highest power in the lowest harmonic of the syllable. In order to make comparisons between different syllables whose base frequency can vary widely, we convert the pitches into semitones as shown below:

s=12*log2(pitchbaseline)

where *s* is the change in pitch in semitones, *pitch* is the observed pitch, and *baseline* is the average pitch across the 3 d of baseline for that particular syllable. For all group analysis, the means reported are the means over all birds and over all syllables weighted by the proportion of times they sang each syllable. This was chosen to account for the fact that syllables that are sung more often are exposed a greater number of times to the shifted auditory feedback. Pitch quantification was performed using custom-written scripts in MATLAB (RRID:SCR_001622).

### Error quantification

For each of our groups, we had between four and eight birds, each bird performed between four and 12 different syllables whose pitch could be quantified, and each syllable was repeated between 40 and 600 times per day. As a result, while we have several thousands of data points toward establishing the position of the mean pitch change per group for each day, the structure of the data is hierarchical and error accumulates at different levels (birds, syllables and iterations). Grouping all the data together and estimating the standard error of the mean underestimates the error by ignoring the non-independence between data points due to the hierarchical structure. On the other extreme, aggregating points and simply using individual birds or syllables does not allow us to use all of our data effectively. This is a complex problem that different studies, including our own prior efforts have used varying methods to address (Galbraith et al., 2010; Sober and Brainard, 2012; Aarts et al., 2014; Tian and Brainard, 2017). To more accurately quantify the error in our groups and better account for the variance arising from finite data samples, we use a hierarchical bootstrapping approach (Crowley, 1992; Efron and Tibshirani, 1994). In its simplest form, bootstrapping involves generating *N* (*N* = 10^4^ throughout this paper) random subsamples of the dataset by sampling with replacement from the original data and computing a metric of interest for each subsample. This results in having a distribution of the metric of interest, the 67% confidence interval of which provides an accurate estimate of the uncertainty in measurement of that metric in the original dataset (Efron, 1981, 1992; Efron and Tibshirani, 1994). For example, if one wanted to obtain the uncertainty in measuring the kurtosis of the data, one would generate bootstrap subsamples and calculate the kurtosis for each subsample. The standard deviation of the population of kurtosis values so obtained gives an accurate estimate of the uncertainty of the kurtosis in the original data. In the special case of estimating a population of means (which is the metric of interest in all instances in this paper), the uncertainty in measurement referred to above corresponds to the standard error of the mean of the dataset. However, bootstrapping by itself does not solve the problem of non-independence in hierarchical data. Crucially, to address this issue the resampling described above has to be done separately over each level of the hierarchy. This means that to generate a single subsample, we first resampled among the birds, then for each selected bird, we resampled among its syllables and finally for each syllable, we resampled among its iterations. Finally, we acknowledged that Bengalese finches can vary greatly in their syllable repertoires from one bird to the next. While all birds typically have an order of 10 syllables, some birds repeat one or two syllables with a much higher frequency than any other syllable while others represent each syllable equally. Since the bootstrapping procedure was used to calculate uncertainty of measurement due to sampling from a limited number of birds, we posited that each syllable would be equally likely in hypothetical new birds. Therefore, we set the number of iterations of a particular syllable that could occur in a bootstrapped subsample to be independent of the frequency of occurrence of that syllable in the actual data. All the data for the subsample were then combined and their mean was calculated for the subsample. Note that this procedure only applies to our estimate of measurement uncertainty (not the mean pitch values), since the means reported in the results are calculated from the actual data collected. This process was then repeated N times. In order to also account for the error in estimation of the mean of each syllable during baseline, the resampling was performed on pitch measurements recorded in hertz (Hz) and the measurements were converted to semitones just before calculating the mean pitch for each subsample. A similar procedure was followed for quantifying error during washout. To account for the error in estimation of pitch on the last day of pitch shift, the subtraction of the mean pitch on the final day of shift through the washout period was performed following the resampling. Our error quantification was performed using custom written scripts in MATLAB (all analysis scripts will be made available on GitHub postpublication; https://github.com/soberlab/Dopamine_Headphones_Paper_code).

### Hypothesis testing with bootstrap

In addition to using bootstrapping to compute error estimates as described above, we also used a bootstrapping approach to test whether vocal pitches were significantly different across time or experimental conditions by computing direct posterior probabilities for individual hypotheses. Hence, we report our results in terms of direct probabilities of a sample being greater than or equal to another sample or fixed value in lieu of *p* values. Specifically, we resample the distribution for each group and calculate the mean 10^4^ times to produce a distribution of resampled means to calculate the variance associated with having a finite number of samples.

These resampled distributions were used to compute whether the two distributions of vocal pitches were significantly different. For all instances in this paper, we use two-way tests with α = 0.05. This means that a probability is significant if the probability supporting the hypothesis, *p* < α/2 or if *p* > (1 – α/2), i.e., if *p* < 0.025 or if *p* > 0.975. In the case of computing the probability of the mean of a group being different from a constant, one can calculate the proportion of the population of bootstrapped means (as defined above, Error quantification) being greater than or equal to said constant. For example, to compute the probability that the mean pitch of a particular group is significantly different from zero, one would compute the proportion of the population of bootstrapped means that are greater than or equal to zero. If this proportion is <0.025 then the pitch of the group of interest is significantly below zero while if the proportion is >0.975 then the pitch of the group is significantly above zero.

We used a similar approach to compute significant differences between two groups of interest. In this case, we compute a population of bootstrapped means for each group. From these two bootstrapped populations, we compute a joint probability distribution between the bootstrapped means of the two groups. The null hypothesis representing no difference between the two groups would correspond to a circle centered about the unity line. Therefore, to test the difference between the two groups, we compute the volume of the joint probability distribution on one side of the unity line (including the unity line itself) to quantify the probability of one group being greater than or equal to the other group. If the probability computed is >0.975, then the first group is statistically greater than the second group. Alternatively, if the probability computed is <0.025, then the first group is statistically less than the second group. We computed multiple comparisons between groups by computing differences between two groups at a time and applied a Bonferroni correction to the threshold for significance. Our statistical tests were performed using custom scripts written in MATLAB which will also be made available on GitHub postpublication; https://github.com/soberlab/Dopamine_Headphones_Paper_code.

### Validating our results with linear mixed models (LMMs)

To ensure that our results were robust to our choice of error quantification and design, we also separately reported frequentist statistical tests on our results. Since our data are hierarchical (see above, Error quantification), the recommended way to perform frequentist statistics on our data are through LMMs (Aarts et al., 2014, 2015). Specifically, we built LMMs by using bird identity and syllable identity within a bird as variable effects and tested for significance of fixed effect factors. Concretely, our LMMs were of the form:

$$Pitch_{ijk}=\beta_{0jk}+\beta_{1}*x_{ij}+\varepsilon_{ij}$$

$$\beta_{0jk}=\beta_{00k}+b_{0jk}$$

$$\beta_{00k}=\beta_{000}+c_{00k}$$

where x_ij_ refers to the condition of the shift (±1 semitone or 0 semitone) and is the fixed effect while b_0jk_ accounts for the bird identity and c_00k_ accounts for syllable identities within a bird which are both variable effects. The code for hypothesis testing using LMMs was also done in MATLAB and will be available on GitHub postpublication; https://github.com/soberlab/Dopamine_Headphones_Paper_code.

## Results

We performed pitch shift experiments on six unlesioned birds (three each for upward shifts and downward shifts) and eight lesioned birds (four for upward pitch shift and four for downward pitch shift). Following the end of the pitch shift, we also collected data during the washout period, i.e., when the pitch shift is set back to zero and the bird typically reverts its pitch back to baseline. All six unlesioned birds had washout data collected for 6 d following the end of shift. Of the eight 6-OHDA lesioned birds, four had data for washout for 7 d each (we were unable to record washout data for the other four lesioned animals due to technical problems associated with long-term use of the headphones). In addition, we performed control experiments with two unlesioned birds fitted with headphones who heard unshifted (zero pitch shift) auditory feedback and eight birds who received 6-OHDA lesions but did not undergo any pitch shifts (for complete details, see Materials and Methods).

### 6-OHDA lesions reduce dopaminergic innervation of Area X

**Figure 2.**
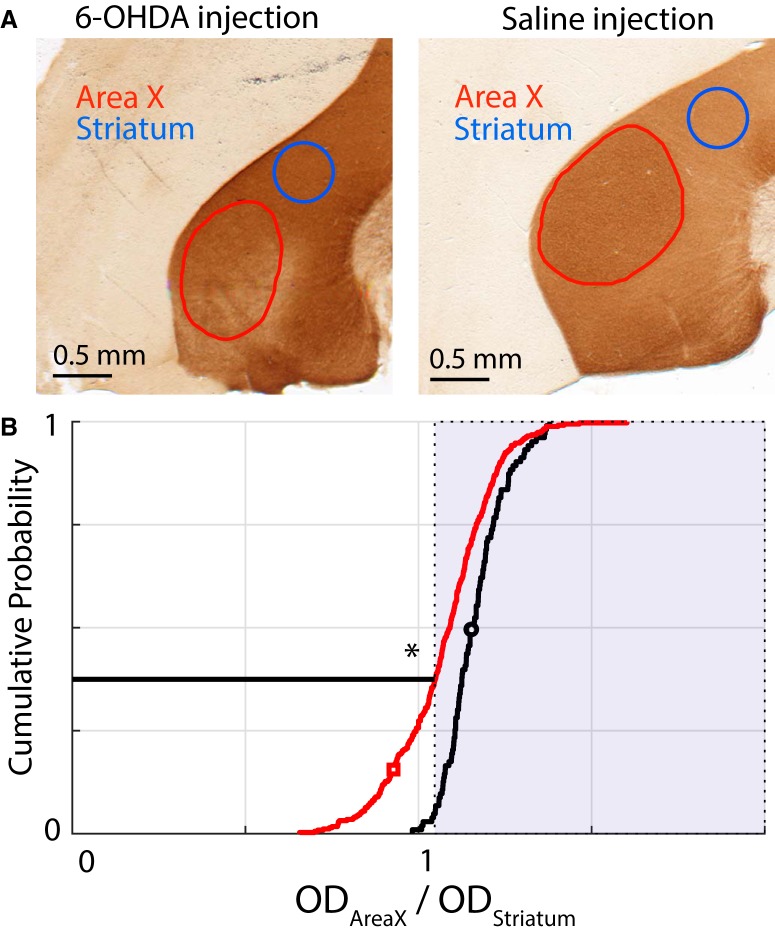
Metric for quantifying the extent of our lesions in our population of birds. We used an OD ratio between Area X and the surrounding basal ganglia (see Materials and Methods) and compared the cumulative ratios between a saline-injected population (*N* = 4 birds) and our 6-OHDA lesioned population (*N* = 16 birds). ***A***, Examples of 6-OHDA lesioned (left) and saline-injected (right) sections. The red trace demarcates the Area X boundary. The blue circle is chosen to represent a uniformly stained section of the rest of the striatum. The ratio for each section is calculated as the OD ratio between these two regions. ***B***, Cumulative distribution plots for the saline-injected birds (black trace) and the 6-OHDA lesioned birds (red trace). The shaded portion represents ratios that are greater than the 5th percentile for the saline-injected birds. By this metric, 37.5% of all 6-OHDA lesioned sections have a smaller OD ratio. The black and red symbols correspond to the examples shown in ***A***. The * represents a statistically significant difference between the red trace and the black trace (Kolmogorov–Smirnov test; *p* < 0.05; see Results for full description).

We quantified the lesion extent using a metric developed as part of our prior work (Hoffmann et al., 2016). Specifically, we used sections of Area X stained with DAB, a chromogen that conjugates to antibodies specific for TH, the rate limiting enzyme involved in catecholamine synthesis and a reliable marker for dopaminergic and noradrenergic innervation (Fig. 2). TH-DAB does not follow the Beer–Lambert law and varies in stain intensity even within the same animal (Van Eycke et al., 2017). As a result, quantification is typically performed between hemispheres within one section comparing a lesioned to an unlesioned hemisphere. However, we had to perform bilateral lesions for our experiments since song learning is not known to be lateralized in Bengalese finches. To quantify lesion extent, we used the fact that Area X has denser dopaminergic innervation and thus stains darker by TH-DAB than the surrounding striatum (Bottjer, 1993; Soha et al., 1996). Specifically, we quantified an OD ratio for a batch of birds that had been injected with saline into Area X (*N* = 4 birds; data reanalyzed from Hoffmann et al., 2016) and produced a cumulative distribution plot of the ratio across all sections for these birds. We then defined the 5th percentile of that distribution as the threshold for defining lesioned sections (see Materials and Methods). When we produced a similar cumulative distribution plot of the OD ratio for all 16 of our 6-OHDA lesioned birds, ∼37.5% of all sections were below the threshold defined above (Fig. 2*B*). This was somewhat smaller than the lesion extent for the cohort of birds in (Hoffmann et al., 2016) in which 50% of lesioned sections were below the threshold. However, the lesions were qualitatively similar between the two groups. In addition, the population of OD ratios for the 6-OHDA lesioned birds was consistently below that for the saline-injected birds as verified by a two-sample Kolmogorov–Smirnov test (K = 0.3467; *p* = 5.75 × 10^−9^). We have also previously shown that such 6-OHDA lesions have no discernible effect on the existing low levels of noradrenergic innervation of Area X (Hoffmann et al., 2016).

### 6-OHDA lesioned birds reduce pitch even in the absence of auditory error

**Figure 3.**
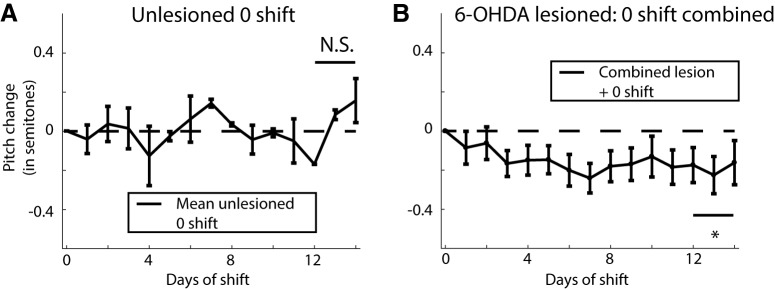
Quantifying the effect of headphones without any pitch shifts on the average change in pitch of the bird with or without lesions. ***A***, Mean change in pitch of song for two unlesioned birds with headphones but no shifts through the headphones (reproduced from Sober and Brainard, 2009, their Supplemental Fig. 6). ***B***, Mean change in pitch for 6-OHDA lesioned birds combining both birds with headphones but no shift in pitch (*N* = 5 birds) or without headphones (*N* = 3 birds) for a total of eight birds. The group averages for the two groups and the individual traces for all eight birds is shown in Extended Data Figure 3-1. N.S. represents not significantly different from zero, while the * represents a significant difference when comparing the last 3 d of shift combined from zero (*p* < 0.05).

We showed earlier that in unlesioned animals, the headphones do not cause changes in vocal pitch in the absence of any shifts in feedback pitch (Sober and Brainard, 2009). As shown in Figure 3*A*, the mean pitch across days 12 through 14 of the experiment for these birds was found to be 0.02 ± 0.07 semitones (all measures of mean pitch reported are mean ± SEM). Since this particular dataset only consists of six data points, it did not make sense to perform a bootstrap analysis (here SEM is measured across six data points; see Materials and Methods). Instead, we used a one sample *t* test and found that this distribution was not significantly different from zero (*t* = 0.35; df = 5; *p* = 0.74).

10.1523/ENEURO.0190-19.2019.f3-1 Extended Data Figure 3-1 ***A***, Mean change in pitch for 6-OHDA lesioned birds either with headphones but no shift in pitch (black trace; *N* = 5 birds) or without headphones (gray trace; *N* = 3 birds). ***B***, Mean change in pitch for individual lesioned birds subjected to zero pitch shift either with or without headphones. Download Figure 3-1, EPS file.

Data from eight birds with 6-OHDA lesions but without any pitch shift revealed an unexpected systematic lowering of vocal pitch after dopamine depletion. Of those, five birds had headphones that conveyed unshifted auditory feedback (i.e., no pitch shift) and three birds had no headphones attached. When we analyzed the mean pitch change for each day for these two groups, we found them within error bars of each other for all 14 d of the experiment, and their pitch change across days 12 through 14 (–0.20 ± 0.14 with headphones; –0.16 ± 0.06 without headphones) were statistically indistinguishable (probability of resampled mean pitch with headphones greater than that without headphones was *p* = 0.098; see Materials and Methods, Hypothesis testing with bootstrap). As a result, we combined the data from the two groups to compute the mean shift in pitch over the course of the experiment as shown in Figure 3*B* (the means for individual groups and traces for individual birds are shown in Extended Data Fig. 3-1). The overall shift in pitch over days 12 through 14 for this combined group was –0.19 ± 0.08 semitones. This decrease in pitch was statistically significant (probability of resampled mean pitch greater than or equal to zero was *p* = 0.0029), demonstrating, unexpectedly, that 6-OHDA lesions of Area X impacted song production by reducing the average pitch over time even in the absence of pitch-shifted auditory feedback.

### 6-OHDA lesioned birds do not respond adaptively to pitch-shifted auditory error

**Figure 4.**
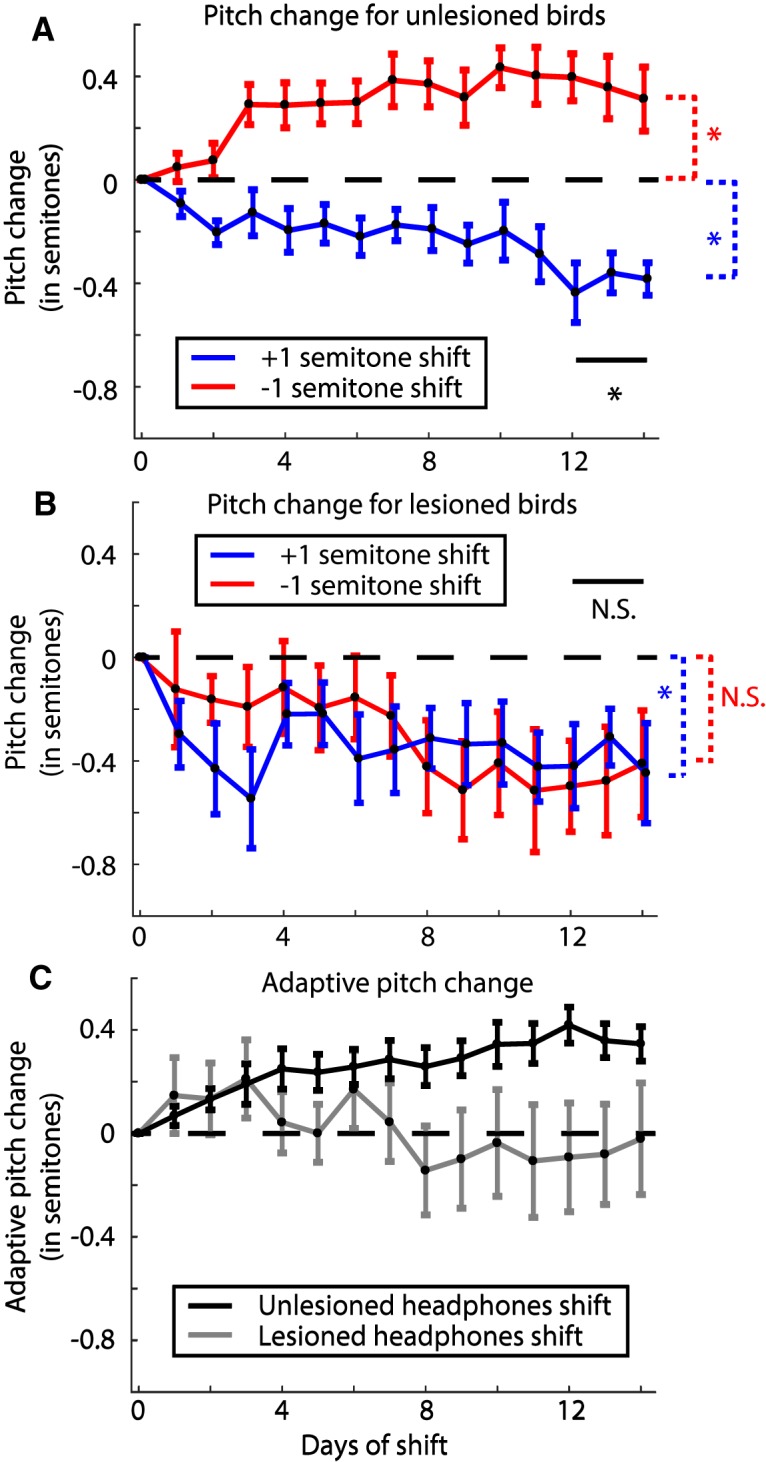
Change in pitch in response to pitch shift errors through the headphones in unlesioned and 6-OHDA lesioned birds. ***A***, Change in pitch from baseline over the period of pitch shift for unlesioned birds broken up by the direction of introduced shift in pitch (data reanalyzed from Sober and Brainard, 2009). The graph shows that birds increase their pitch over time in response to a downward pitch shift (red trace; *N* = 3 birds) and decrease their pitch to an upwards pitch shift (blue trace; *N* = 3 birds). Traces for individual birds are shown in Extended Data Figure 4-1*A*. ***B***, Same graph as in ***A*** quantified for 6-OHDA lesioned birds (*N* = 4 birds for each trace). Individual birds are shown in Extended Data Figure 4-1*B*. ***C***, Adaptive change in pitch (see Results) for unlesioned birds (black trace; *N* = 6 birds) and 6-OHDA lesioned birds (gray trace; *N* = 8 birds). For ***A***, ***B***, the * and N.S. in black represent significant and not significant differences, respectively, between the two shift conditions, while the color coded differences check difference of each group from zero (see Results; Table 1).

In unlesioned animals, birds respond to a pitch shift through the headphones in an adaptive manner. Specifically, when subjected to a +1 semitone pitch shift through the headphones, the unlesioned birds compensate adaptively by lowering their pitch (mean pitch change over days 12–14 for *N* = 3 birds was –0.40 ± 0.07 semitones; probability of resampled mean pitch greater than or equal to zero was *p* < 10^−4^; limit due to resampling 10^4^ times; Fig. 4*A*, blue trace) and when subjected to a –1 semitone shift in pitch, the unlesioned birds increase their pitch [mean pitch change over days 12–14 for *N* = 3 birds was 0.36 ± 0.11 semitones (Fig. 4*A*, red trace); probability of resampled mean pitch greater than or equal to zero was *p* = 0.9996, recall that in our bootstrapping analysis we conclude that distributions are significantly different if the probability that one is greater than or equal to the other is <0.025 or >0.975 (see Materials and Methods); traces for individual birds are shown in Extended Data Fig. 4-1*A*]. The plot depicting adaptive change in pitch (inverting *y*-axis for +1 semitone shift birds) for unlesioned birds is shown in Figure 4*C*, black trace. A direct comparison between the populations of –1 semitone shift and +1 semitone shift birds revealed a complete non-overlap among posterior distributions of sampled means (probability of resampled mean pitch for +1 semitone shift greater than or equal to that for –1 semitone shift was *p* < 10^−4^; limit due to resampling 10^4^ times). This resampling-based analysis reaffirms our initial finding (Sober and Brainard, 2009) that unlesioned birds respond adaptively to pitch-shifted auditory errors and compensate accordingly for them, despite the fact that this earlier paper did not take into account the hierarchical nature of the data and the resulting propagation of uncertainty when computing statistical significance.

10.1523/ENEURO.0190-19.2019.f4-1 Extended Data Figure Extended Data 4-1 *A*, Mean change in pitch for individual unlesioned birds subjected to a ±1 semitone pitch shift. ***B***, Mean change in pitch for individual lesioned birds subjected to a ±1 semitone pitch shift. Note that one bird subjected to a +1 semitone shift has a discontinuity at shift day 12 since the bird did not sing at all that day. Download Figure 4-1, EPS file.

For 6-OHDA lesioned birds, however, all birds decreased their pitch over time regardless of the direction of pitch shift through the headphones (Fig. 4*B*), similar to what we observed in lesioned birds with no pitch shifts (Fig. 3*B*). The +1 semitone shift group had a final pitch change of –0.38 ± 0.16 semitones (probability of resampled mean pitch greater than or equal to zero was *p* = 0.0040) while the –1 semitone shift group changed to a final pitch of –0.46 ± 0.19 semitones (probability of resampled mean pitch greater than or equal to zero was *p* = 0.0747) relative to the baseline (traces for individual birds are shown in Extended Data Fig. 4-1*B*). The two groups were not statistically different from each other (probability of resampled mean pitch of +1 semitone shift group being greater than or equal to that of –1 semitone shift group was *p* = 0.26). We also compared each group to the no shift group and did not find statistically significant results (probability of resampled mean pitch of no shift group being greater than or equal to that of –1 semitone shift group was *p* = 0.62; probability of resampled mean pitch of no shift group being greater than or equal to that of +1 semitone shift group was *p* = 0.91). All statistical comparisons have been summarized in Table 1. Furthermore, when we quantified the adaptive change in pitch for this group, the final change in pitch was close to zero (Fig. 4*C*, gray trace). This suggests that following 6-OHDA lesions, birds do not respond adaptively to the auditory error. Instead, the birds seem to reduce their pitch over time regardless of the direction or presence of pitch-shifted auditory error. Note that as was mentioned above and shown in Table 1, there was not a statistically significant difference between the Lesioned –1 semitone shift group and zero. This was due to the fact that while birds subjected to the –1 semitone shift did reduce their pitch on average, a few syllables for each bird increased their pitch, resulting in a group effect that fell short of significance. Since our error quantification treats the contribution from each syllable equally, the effects of individual syllables add up resulting in a not statistically significant difference (see Materials and Methods, Error quantification).

**Table 1.** Statistical tests summary

Hypothesis tested, Bayesian probability of group on left being >= column heading (see Materials and Methods, Hypothesis testing with bootstrap)			
Groups compared	Zero	Lesioned +1 semitone shift	Lesioned –1 semitone shift
Lesioned 0 shift	**0.0029**	0.91	0.62
Lesioned +1 semitone shift	**0.0040**		0.26
Lesioned –1 semitone shift	0.0747		

Results of statistical tests for ±1 semitone shift and 0 shift lesioned groups. The probabilities for each hypothesis are reported by testing the probability of the group on the left being greater than or equal to the various column headings. Blank spaces represent tests that either do not make sense to make or have been reported on another row. The probabilities that are statistically significant at α = 0.05 are depicted in bold.

Since the hierarchical bootstrapping as we have performed here to calculate statistical tests and standard errors has not been widely applied to such datasets in neuroscience previously, we also analyzed our data using hierarchical LMMs (Aarts et al., 2014, 2015). LMMs have been widely applied to datasets involving large numbers of samples from a small number of subjects such as non-human primate studies (Arlet et al., 2015; Pleil et al., 2016) and rodent studies (Liang et al., 2015) or to analyze repeated measures or time series data (Wykes et al., 2012; Howe et al., 2013). Specifically, we built LMMs to test the effects of the shift condition while controlling bird identity and specific syllables within each bird as variable effects (see Materials and Methods, Validating our results with linear mixed models). For the unlesioned birds the LMM revealed a strong effect of the shift condition (*t* = 7.17; *p* = 7.92 × 10^−13^) on final pitch at the end of the shift period. For the 6-OHDA lesioned birds, the effect of the shift condition (+1 semitone shift vs –1 semitone shift versus no shift) was not significant (*t* = 1.91; *p* = 0.056). Also, when we combined the shift groups and compared them to the no shift groups, the effect was not statistically significant (*t* = 1.47; *p* = 0.14). That these models give us the same statistically significant results as our bootstrapping procedure gives us an independent verification of our error calculation and statistics.

### No correlations between lesion extent and changes in pitch

We measured the extent of 6-OHDA lesions by quantifying the proportion of histologic sections that fell below the 5th percentile of section OD ratio for saline-injected birds (see Materials and Methods). We can use this same threshold to obtain a rough metric of the lesion extent for each bird. Using this lesion extent, we computed correlations between the lesion extent and a variety of metrics of changes in pitch during the experiment and these have been summarized in Table 2.However, we saw no significant correlations.

**Table 2.** Correlations between lesion extent and changes in song metrics

Lesion extent versus	Pearson’s correlation, *r*	Correlation significance, *p*
Final pitch change	0.4261	0.1466
Baseline variance	0.296	0.3261
Final variance	–0.0498	0.8716
Percent increase in variance	–0.4272	0.1454

The lesion extent for each bird was defined as the proportion of sections with OD ratio below the 5th percentile of OD ratios for the population of saline-injected birds. A Pearson’s correlation coefficient (*r*) and the associated *p* value is reported for this lesion extent versus changes in song metrics. Variances were computed across either 3 d of baseline or the final 3 d of the shift period.

### Washout is impaired by dopamine depletion

**Figure 5.**
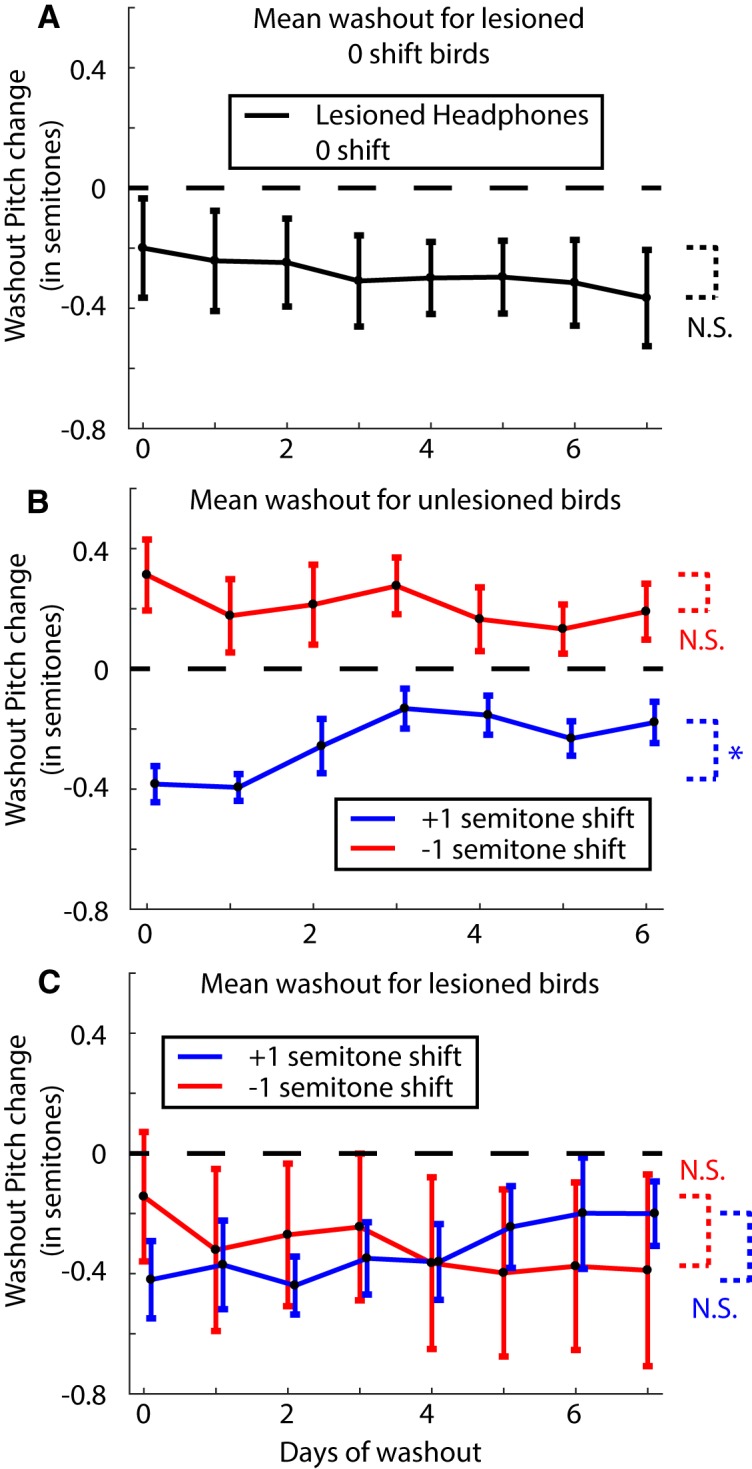
Analysis of change in pitch during washout for lesioned and unlesioned birds. ***A***, Mean change in pitch during washout for lesioned birds with headphones but no pitch shift (*N* = 5 birds). Day 0 refers to the last day of the shift period. Pitch shift is turned off at the end of this day. Individual bird traces are shown in Extended Data Figure 5-1*A*. ***B***, Mean change in pitch during washout for unlesioned birds (*N* = 3 birds for each trace). Individual bird traces are shown in Extended Data Figure 5-1*B*. ***C***, Mean change in pitch during washout for 6-OHDA lesioned birds (*N* = 2 birds for each trace). The extremely large error bars are due in part to the bimodal nature of the data (see individual birds in Extended Data Fig. 5-1*C*). The statistical tests check the last 3 d of the shift period against the last 2 d of washout with * representing a significant difference (*p* < 0.05) and N.S. representing not significant (see Results for full tests).

**Figure 6.**
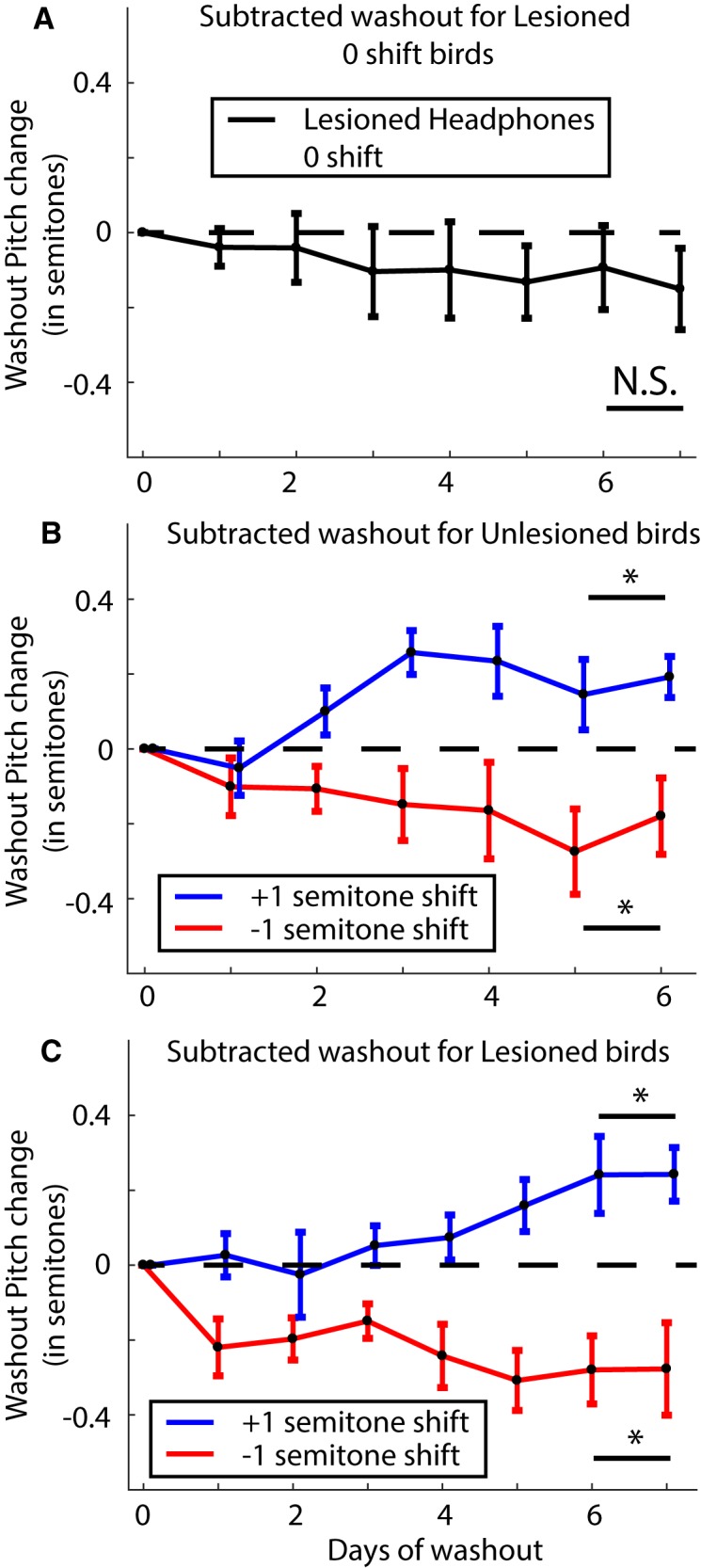
Results when measuring the dynamics of the change in pitch or Δ(Pitch) during washout by subtracting out the pitch change on the last day of shift through the washout period. ***A***, Δ(Pitch) during washout for lesioned no shift birds (*N* = 5 birds). ***B***, The same analysis as in ***A*** for unlesioned birds subjected to ±1 semitone shift (*N* = 3 birds each). ***C***, The same analysis as in ***A*** for lesioned birds subjected to ±1 semitone shift (*N* = 2 birds each). The * and N.S. refer to a significant difference versus not, respectively, for each group compared to zero over the last 2 d of washout.

Following the end of the shift period, we turned the pitch shift through the headphones back to zero and recorded the birds’ songs for an additional 6–7 d. During this period, birds without lesions typically revert their pitch back toward baseline levels (Sober and Brainard, 2009). Hence, we refer to this period as washout. We first collected washout data from the birds that had 6-OHDA lesions and headphones but no shifts. As stated earlier, by days 12 through 14 of the shift period, these birds had a mean pitch of –0.20 ± 0.13 semitones. By days 6 and 7 of the washout period, their pitch had changed to –0.34 ± 0.15 semitones (Fig. 5*A*; traces for individual birds are shown in Extended Data Fig. 5-1*A*). The probability of the resampled mean pitch during the end of the shift period being greater than or equal to that during the end of the washout period was *p* = 0.67. Therefore, although the change was not statistically significant, the mean pitch did drop further during washout. In order to quantify how much the pitch changes in response to the end of the sensory perturbation (pitch shift), we subtracted the mean pitch for each syllable on the last day of pitch shift throughout the entire washout period and quantified the resulting deviation in pitch (Fig. 6*A*). This emphasizes the dynamics of how the pitch changes or Δ(Pitch) over time during washout in response to the end of the shift. The resulting change in pitch was found to be –0.12 ± 0.11 semitones (probability of resampled mean pitch greater than or equal to zero was *p* = 0.22).

10.1523/ENEURO.0190-19.2019.f5-1 Extended Data Figure 5-1 Washout traces for individual birds. ***A***, Individual birds that had a 6-OHDA lesion, with headphones but no pitch shift. Each color is a separate bird. ***B***, Washout traces for individual birds that were unlesioned and subjected to a ±1 semitone pitch shift. ***C***, Washout traces for individual 6-OHDA lesioned birds subjected to a ±1 semitone pitch shift. Download Figure 5-1, EPS file.

Unlesioned birds displayed a robust return to baseline following the end of the pitch shift period as shown in Figure 5*B* (see traces for individual birds in Extended Data Fig. 5-1*B*). For birds subjected to a –1 semitone shift, they reduced their pitch from 0.36 ± 0.11 semitones at the end of shift to 0.17 ± 0.08 semitones during the last 2 d of washout (probability of mean resampled pitch during washout being greater than or equal to that at the end of shift was *p* = 0.08). Equivalently, birds subjected to a +1 semitone shift increased their pitch from –0.40 ± 0.07 semitones at the end of the shift period to –0.20 ± 0.05 semitones by the end of the washout period (probability of mean resampled pitch during washout being greater than or equal to that at the end of shift was *p* = 0.98). We also computed the dynamics underlying the Δ(Pitch) over time during the washout period by subtracting the pitch for each syllable on the last day of shift through the washout period (Fig. 6*B*). Birds subjected to a +1 semitone shift, having reduced their pitch during the shift increased their pitch during washout. The last 2 d of washout had a mean change relative to the last day of shift of 0.17 ± 0.07 semitones (probability of resampled mean pitch lesser than or equal to zero was *p* = 0.0003). Similarly, birds subjected to a –1 semitone shift reduced their pitch back toward baseline during washout by –0.22 ± 0.11 semitones relative to the last day of shift (probability of resampled mean pitch greater than or equal to zero was *p* = 0.0064).

For our 6-OHDA lesioned birds, only four out of eight birds had data for 7 d of washout due to difficulties in keeping the headphones attached (two each for upward and downward shifts). We repeated the analysis for washout for these birds as described above for lesioned no shift and unlesioned birds. First, the mean change in pitch from the last day of shift through the washout period is shown in Figure 5*C*. Birds subjected to a +1 semitone shift returned their pitch back toward baseline increasing their pitch from –0.31 ± 0.19 semitones at the end of the shift period to –0.20 ± 0.14 semitones by the end of the washout period (probability of mean resampled pitch during washout being greater than or equal to that at the end of shift was *p* = 0.75; Fig. 5*C*, blue trace). Contrary to expectations however, the birds subjected to a –1 semitone shift drifted further away from baseline reducing their pitch from –0.16 ± 0.22 semitones at the end of the shift to –0.38 ± 0.30 semitones by the end of the washout period (probability of mean resampled pitch during washout being greater than or equal to that at the end of shift was *p* = 0.35; Fig. 5*C*, red trace). The traces for individual birds are shown in Extended Data Figure 5-1*C*.

Curiously, when we quantified the change in pitch in response to the end of the sensory perturbation subtracting the pitch change through the last day of shift through the washout period as before (i.e., measured the direction of pitch changes during washout, without considering the magnitude or direction of the pitch changes at the end of the shift period), the dynamics of the change in pitch was very similar to that seen in unlesioned birds (Fig. 6*C*). Lesioned birds subjected to a +1 semitone shift, averaging across the last 2 d of washout, shifted their pitch 0.24 ± 0.06 semitones with respect to the last day of shift (probability of resampled mean pitch lesser than or equal to zero was *p* = 0.0003). Lesioned birds subjected to a –1 semitone shift on the other hand, changed their pitch by –0.28 ± 0.11 semitones with respect to the last day of shift (probability of resampled mean pitch greater than or equal to zero was *p* = 0.0182). This result once again shows the dual effects we are observing following dopamine depletion. First, while not statistically significant, the pitch continued to drop for birds with unshifted auditory feedback. Second, washout was severely impaired in lesioned birds as evidenced by the fact that the birds subjected to a –1 semitone shift drifted further away from the baseline following the end of the shift instead of back toward baseline. Confusingly though, both lesioned and unlesioned birds followed the same dynamics for the Δ(Pitch) over time following the end of the pitch shifted auditory feedback.

## Discussion

Our results reveal two key effects of dopamine manipulation on the control of birdsong. First, all birds subjected to a 6-OHDA lesion of Area X displayed a drop in average vocal pitch which appeared between a week and two weeks after lesion (Figs. 3*B*, 4*B*). Second, 6-OHDA lesioned birds displayed a severe deficit in sensorimotor learning as is evidenced by the lack of difference in response to a +1 or –1 semitone shift in pitch (Fig. 4*B*,*C*, gray trace).

While our primary finding seems to be one that implicates a role for dopamine in motor production, i.e., ability to produce higher pitched renditions of syllables in a bird’s repertoire, there is also a clear role for dopamine in learning the adaptive response to a sensory perturbation. It is true that when subjected to a +1 semitone pitch shift, there was no difference in mean change of pitch between lesioned (–0.38 ± 0.16 semitones) and unlesioned (–0.40 ± 0.07 semitones) birds (Fig. 4*A*,*B*, blue traces). However, when subjected to a –1 semitone pitch shift, while the adaptive response would be to raise their pitch, lesioned birds lowered their pitch (Fig. 4*B*, red trace). In addition, even for the lesioned birds subjected to a +1 semitone shift, their final change in pitch was not statistically different from the pitch drift seen in lesioned birds with no pitch shift (compare Fig. 3*B*, black trace, and Fig. 4*B*, blue trace). This impairment in sensorimotor learning is reminiscent of deficits in learning in persons with Parkinson’s disease (Paquet et al., 2008; Mollaei et al., 2013) and rodent models of dopamine depletion in striatum and motor cortex (Shiotsuki et al., 2010; Hosp et al., 2011; Hosp and Luft, 2013). Hence our results suggest two factors at play, namely, motor production and sensorimotor learning. Disentangling these has been a hard problem in neuroscience (Beninger, 1983; Wise, 2004) since manipulations that affect motor learning also degrade motor production, complicating efforts to isolate learning mechanisms (Ungerstedt, 1968; Iancu et al., 2005; Cenci and Lundblad, 2007). Here, we isolated the lesions’ effects on motor production by including the lesioned no shift group.

We have previously reported that 6-OHDA lesions of Area X do not produce any changes in number of songs produced or in any general motor behavior (Hoffmann et al., 2016). We similarly did not observe any qualitative difference in song quality or motor behavior between lesioned birds reported in this study and the birds reported in the 2016 study except the systematic drop in average pitch of songs sung after lesion. Note however that the lesioned birds reported in this study were recorded from for two to three weeks longer after lesion than those from the 2016 study due to differences in time required to complete the behavioral experiments after lesion. It therefore seems likely that this extended timeframe was necessary to observe the aforementioned pitch drop.

Vigor has been characterized as motivation (Salamone et al., 2007; Salamone and Correa, 2012), speed of movements, or both (Mazzoni et al., 2007; Turner and Desmurget, 2010). A reduction in motor vigor following dopamine depletion could explain the systematic drop in pitch we observed. Dopamine has been shown to be associated with vigor in humans and other mammalian systems (Niv et al., 2007; Beierholm et al., 2013; Panigrahi et al., 2015; Berke, 2018). In our experiments, we found that following 6-OHDA lesions of Area X the average pitch across all syllables for each bird dropped by roughly 11–13 d after lesion. Higher pitched syllables require a combination of greater muscle activation and higher air sac pressure to be produced (Goller and Suthers, 1996; Elemans et al., 2008, 2015; Riede et al., 2010), suggesting that higher pitched renditions of a particular syllable are more effortful to produce than lower pitched ones. We thus hypothesize that while unlesioned birds are capable of flexibly changing their pitch in a bidirectional fashion, dopamine lesioned birds will display a deficit in raising their pitch due to the increased effort required to do so. A related observation supporting our interpretation of our results is that birds sing at an elevated pitch when singing directed songs to females (Sakata et al., 2008; Leblois et al., 2010). Since it has also been reported that dopamine levels in Area X are elevated during directed song (Sasaki et al., 2006), this fits with the overall trend in our results.

Studies that have targeted individual syllables for pitch changes following dopamine depletions have not reported a systematic drop in pitch after lesion (Hoffmann et al., 2016; Hisey et al., 2018). Our study does not necessarily contradict these results since those studies reported a deficit in learning after lesion by either combining upwards and downwards shifts (Hoffmann et al., 2016) or only driving pitch changes in one direction (Hisey et al., 2018). Additionally, for the birds reported in this study, while the average pitch across all syllables for each bird dropped, some individual syllables did increase their pitch. Furthermore, as noted above the birds in the present study were recorded for a longer period of time after lesion than those reported previously.

The results from our washout data from the 6-OHDA lesioned birds are challenging to interpret. It is true that the lesioned birds subjected to a +1 semitone shift did return their pitch toward baseline and washout seemed to be unaffected for these birds (Fig. 5*C*, blue trace). Previous studies have reported that washout was not affected by dopamine depletion in tasks where birds shifted the pitch of a single syllable to avoid distorted auditory feedback (Hoffmann et al., 2016; Hisey et al., 2018). However, the birds subjected to a –1 semitone shift reduced their pitch resulting in their mean pitch moving further away from the baseline pitch (Fig. 5*C*, red trace). This suggests that washout is severely impaired in dopamine depleted birds. On the other hand, curiously, the change in pitch over time analyzed during washout in response to the end of the shift period was very similar between lesioned and unlesioned birds (compare Fig. 6*B*,*C*). We speculate that the lesion effects reported above could reflect either an inability to adaptively modulate motor output in response to error signals or from miscalculations in computing the error in the first place.

Adaptive sensorimotor learning in songbirds in response to induced auditory pitch shifts has been an effective paradigm to study the computational principles underlying sensorimotor learning (Sober and Brainard, 2009, 2012; Kelly and Sober, 2014). Bayesian inference works well to explain how unlesioned birds respond to auditory errors based on their prior experience of singing (Hahnloser and Narula, 2017; Zhou et al., 2018). However, since 6-OHDA lesioned birds exhibit drops in vocal pitch regardless of the direction of feedback pitch shift, any model that performs an adaptation to an error signal will fail to replicate the data without an additional mathematical mechanism to drive pitch downward in the presence of a reduced dopamine signal. One potential modification to the model would be to add a “relaxation state” into which the system relaxes in the absence of dopamine (Shadmehr and Arbib, 1992; Shadmehr and Mussa-Ivaldi, 1994). However, apart from the mean pitch, which did drop consistently across groups following 6-OHDA lesions, we did not find any other consistent relationships among other moments such as variance, skewness and kurtosis or overall probability distributions of produced pitch that could be used to constrain a revised Bayesian model to explain our results. Future work might therefore investigate the hypothesis that dopamine lesions disrupt sensorimotor learning by degrading the brain’s ability to perform Bayesian inference.

To conclude, our experiments show that dopamine plays a critical role in the brain’s ability to modulate vocal production in response to auditory errors. Future experiments will focus on disentangling specific roles for dopamine in sensorimotor learning by manipulating the dopamine signal at a faster temporal resolution. Results from such experiments could help fill gaps regarding the roles of tonic and phasic dopamine (Grace, 1991) for example and the timeline of error correction. Eventually, results from such experiments can be used to impose mathematical constraints on a computational model detailing the quantitative role of dopamine in such sensorimotor learning.

## Synthesis

Reviewing Editor: Darcy Kelley, Columbia University Decisions are customarily a result of the Reviewing Editor and the peer reviewers coming together and discussing their recommendations until a consensus is reached. When revisions are invited, a fact-based synthesis statement explaining their decision and outlining what is needed to prepare a revision will be listed below. The following reviewer(s) agreed to reveal their identity: NONE. Note: If this manuscript was transferred from JNeurosci and a decision was made to accept the manuscript without peer review, a brief statement to this effect will instead be what is listed below.

The author's have been responsive to the reviews of a previous version of the manuscript transferred from J. Neurosci, The implanted headphones paradigm should be of interest to researchers in the birdsong fiels, the statistical analyses are thorough and thoughtful and the revised manuscript a useful contribution to the literature.
